# Supplementary material for: Structural basis for the recognition of complex-type N-glycans by Endoglycosidase S
Source: Nat Commun. 2018 May 14;9:1874. doi: 10.1038/s41467-018-04300-x (PMC5951799; doi:10.1038/s41467-018-04300-x)
Supplement: Supplementary file 1 — Supplementary Information [file 41467_2018_4300_MOESM1_ESM.pdf]

SUPPLEMENTARY INFORMATION

Structural basis for the recognition of complex-type N-glycans by Endoglycosidase S

Trastoy et al.

a

MEEKTVQVQKGLPSIDSLHYLSENSKKEFKEELSKAGQESQKVKEILAKAQQADKQAQE
LAKMKIPEKIPMKPLHGPLYGGYFRTWHDKTSDPTEKDKVNSMGELPKEVDLAFIFHDW
TKDYSLFWKELATKHVPKLNKQGTRVIRTIPWRFLAGGDNSGIAEDTSKYPNTPEGNKA
LAKAIVDEYVYKYNLDGLDVDVEHDSIPKVDKKEDTAGVERSIQVFEEIGKLIGPKGVD
KSRLFIMDSTYMADKNPLIERGAPYINLLLVQVYGSQGEKGGWEPVSNRPEKTMEERWQ
GYSKYIRPEQYMIGFSFYEENAQEGNLWYDINSRKDEDKANGINTDITGTRAERYARWQ
PKTGGVKGGIFSYAIDRDGVAHQPKKYAKQKEFKDATDNIFHSDYSVSKALKTVMLKDK
SYDLIDEKDFPDKALREAVMAQVGTRKGDLERFNGTLRLDNPAIQSLEGLNKFKKLAQL
DLIGLSRITKLDRSVLPANMKPGKDTLETVLETYKKDNKEEPATIPPVSLKVSGLTGLK
ELDLSGFDRETLAGLDAATLTSLEKVDISGNKLDLAPGTENRQIFDTMLSTISNHVGSN
EQTVKFDKQKPTGHYPDTYGKTSLRLPVANEKVDLQSQLLFGTVTNQGTLINSEADYKA
YQNHKIAGRSFVDSNYHYNNFKVSYENYTVKVTDSTLGTTTDKTLATDKEETYKVDFFS
PADKTKAVHTAKVIVGDEKTMMVNLAEGATVIGGSADPVNARKVFDGQLGSETDNISLG
WDSKQSIIFKLKEDGLIKHWRFFNDSARNPETTNKPIQEASLQIFNIKDYNLDNLLENP
NKFDDEKYWITVDTYSAQGERATAFSNTLNNITSKYWRVVFDTKGDRYSSPVVPELQIL
GYPLPNADTIMKTVTTAKELSQQKDKFSQKMLDELKIKEMALETSLNSKIFDVTAINAN
AGVLKDCIEKRQLLKKLGSGKILHNQNVNSWGPITVTPTTDGGETRFDGQIIVQMENDP
VVAKAAANLAGKHAESSVVVQLDSDGNYRVVYGDPSKLDGKLRWQLVGHGRDHSETNNT
RLSGYSADELAVKLAKFQQSFNQAENINNKPDHISIVGCSLVSDDKQKGFGHQFINAMD
ANGLRVDVSVRSSELAVDEAGRKHTKDANGDWVQKAENNKVSLSWDAQLEGGSGGSGNS
GHHHHHHHHH

b

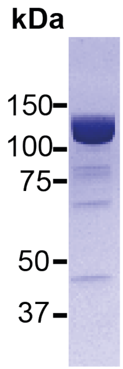

Supplementary Figure 1 Recombinant production of EndoS$_{D233A/E235L}$. **a** The recombinant EndoS$_{D233A/E235L}$ construct (residues 37-999) contains a CPD fusion at the C-terminus (residues 996-1191). **b** SDS-PAGE showing purified EndoS$_{D233A/E235L}$.

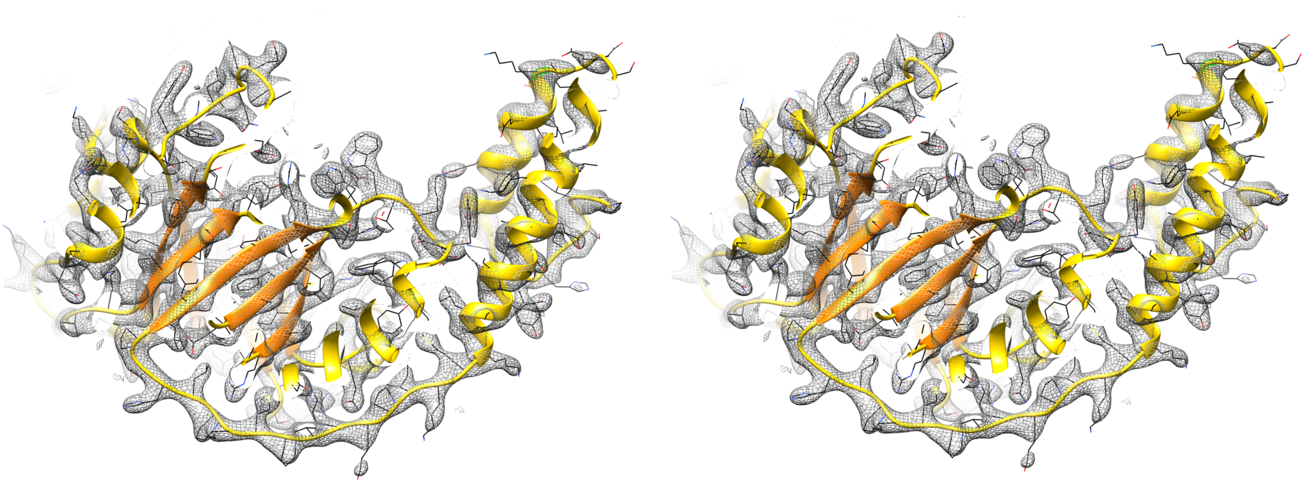

Supplementary Figure 2 Electron density map of the refined $EndoS_{D233A/E235L}$-G2 product complex. Stereo view of the final electron density maps (2mFo-DFc contoured at 1σ) corresponding to the $EndoS_{D233A/E235L}$-G2 product complex.

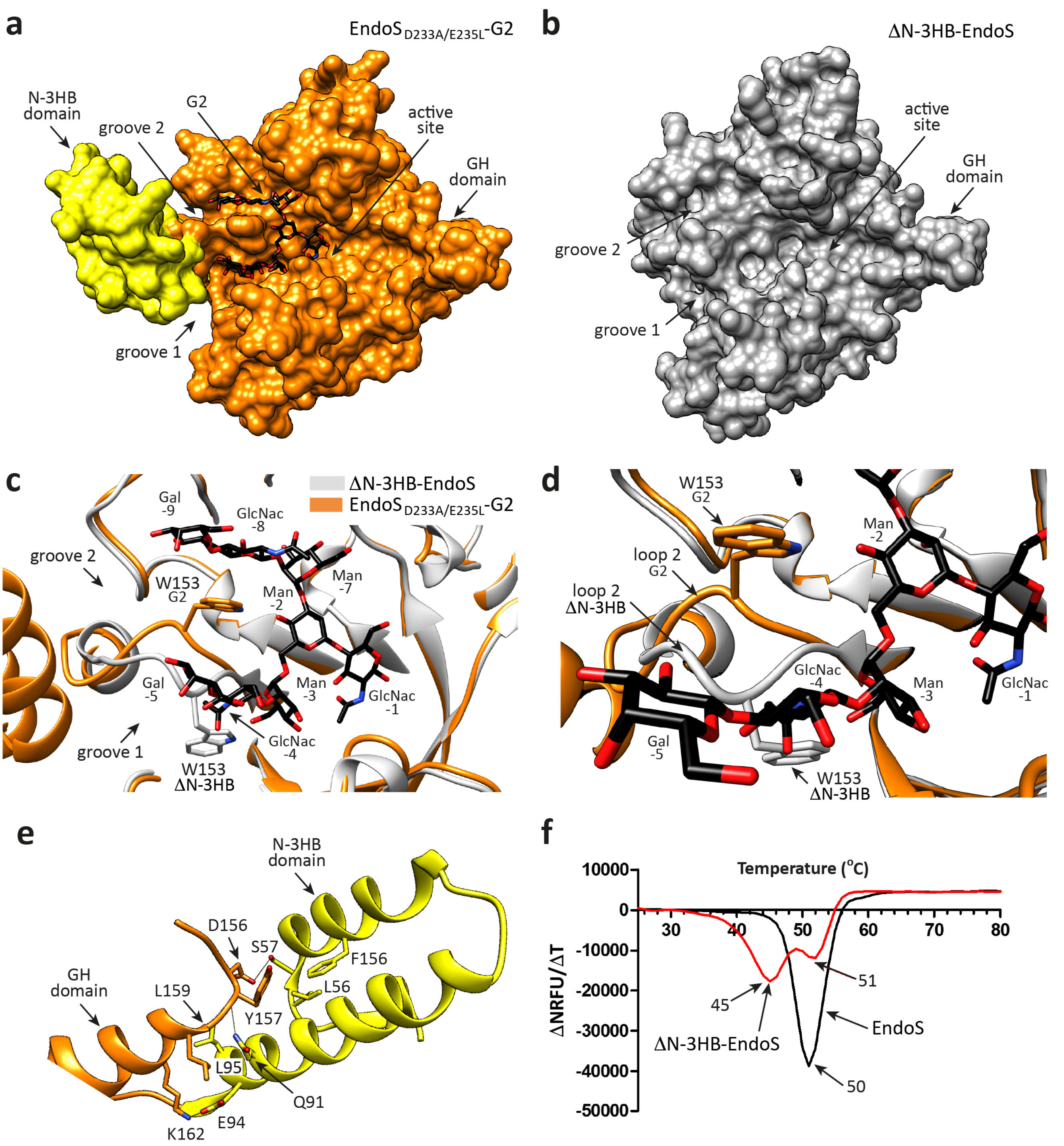

Supplementary Figure 3 Surface representation of **a** EndoS$_{D233A/E235L}$-G2 complex showing N-3HB domain in yellow and the glycosidase domain in orange and **b** ΔN-3HBEndoS in grey. **c** Structural superposition of EndoS$_{D233A/E235L}$-G2 complex (orange) and ΔN-3HBEndoS (grey), showing the conformational arrangement of W153 in loop 2 of the glycosidase domain. **d** Magnified view of panel **c**. **e** Residues of N-3HB domain (yellow) interacting with residues of the glycosidase domain (orange) of EndoS. **f** First derivative of the melting curves of EndoS in black and ΔN-3HBEndoS in red.

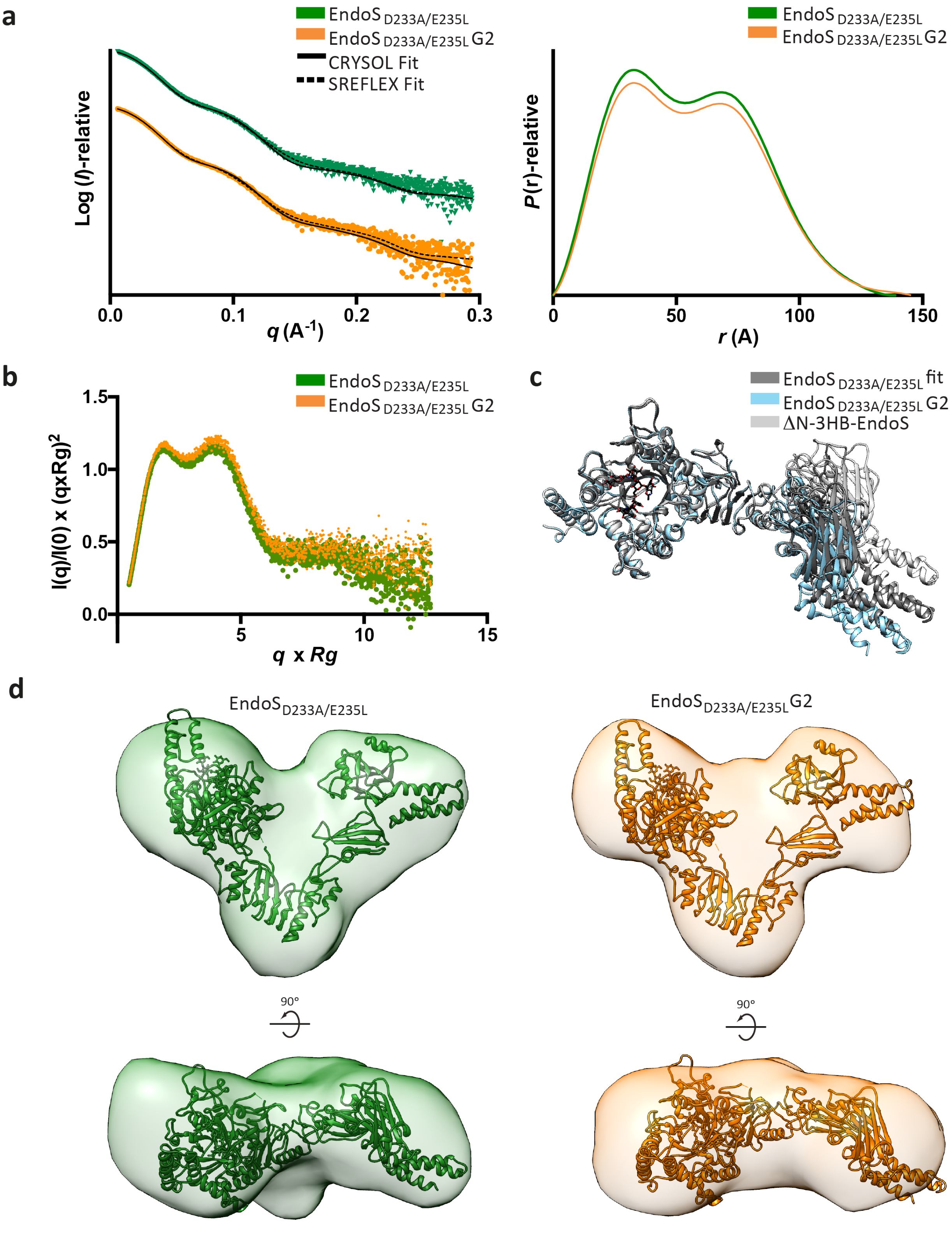

Supplementary Figure 4 Small angle X-ray scattering (SAXS) analysis of $EndoS_{D233A/E235L}$ in the presence and absence of G2 product. **a** On the left panel, scattering curves of $EndoS_{D233A/E235L}$ in absence (green) and presence (orange) of G2 product fitted with the X-ray crystal structure of $EndoS_{D233A/E235L}$-G2 complex (black line) and the best model obtained by SREFLEX; on the right panel, *P(r)* functions distributions of $EndoS_{D233A/E235L}$ in absence (green) and presence (orange) of G2 product. **b** Normalized Kratky plot of $EndoS_{D233A/E235L}$ in absence (green) and presence (orange) of G2 product. **c** Superimposition of $EndoS_{D233A/E235L}$-G2 complex in blue, ΔN3HB-EndoS (4NUZ) in light grey and best model obtained by SREFLEX in dark grey. **d** *Ab initio* modelling with GASBOR.

Superimposition of EndoS$_{D233A/E235L}$-G2 product complex X-ray crystal structure (orange ribbon) into the envelope of EndoS$_{D233A/E235L}$ (orange) and EndoS$_{D233A/E235L}$-G2 product (grey).

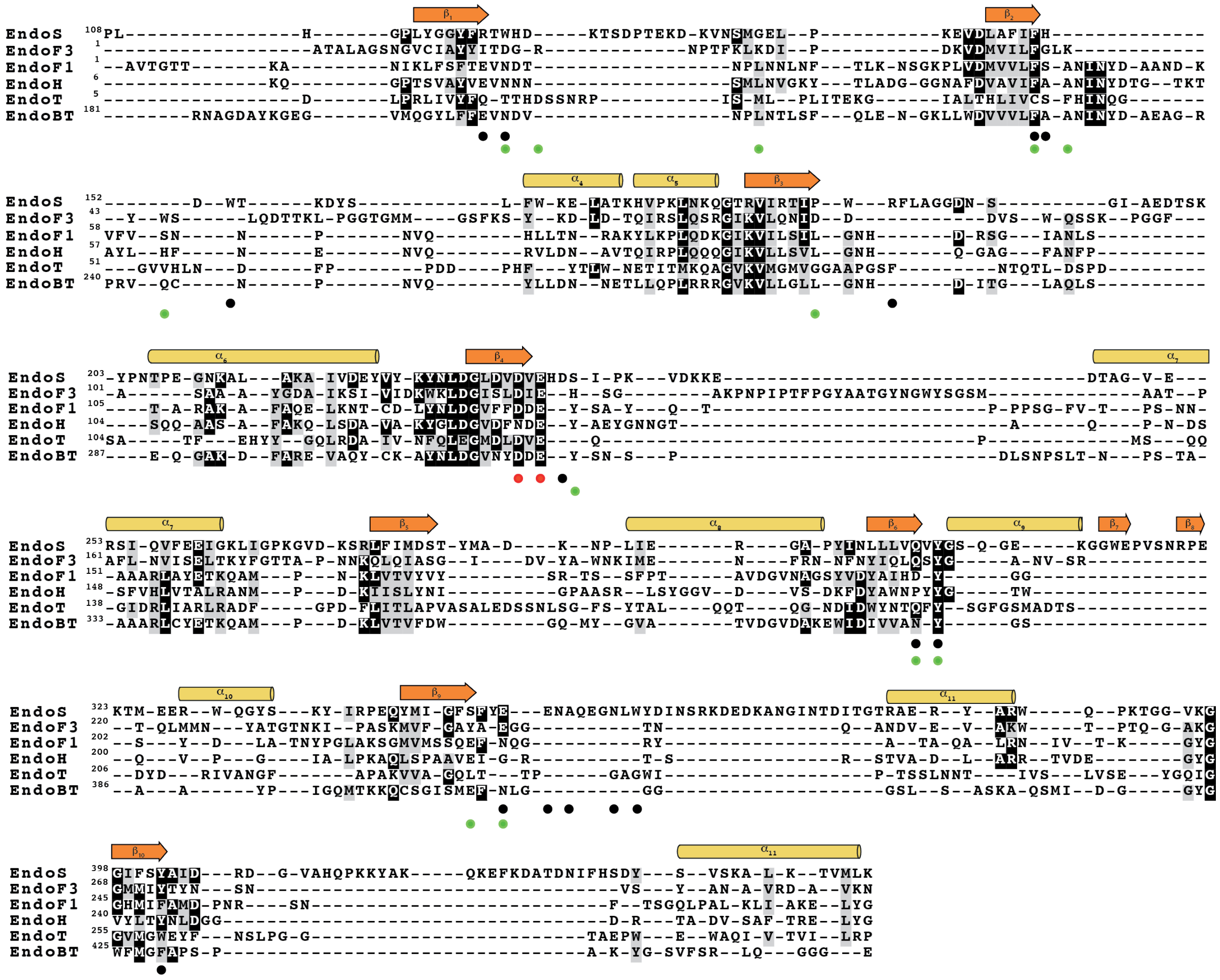

Supplementary Figure 5 Structure based sequence alignment of EndoS with GH18 family enzymes with known endo-*N*-acetyl-β-D-glucosaminidase activity. $EndoS_{D233AE235L}$-G2 product, $EndoF_3$ (1EOM), $EndoF_1$ (2EBN), EndoH (1C3F), EndoT (4AC1) and EndoBT (3POH) were aligned using Chimera. The catalytic residues are marked with red dots and the residues that interact with the G2 product in the crystal structure of $EndoS_{D233AE235L}$-G2 product complex and $EndoF_3$ G2 product complex are marked with black and green dots, respectively.

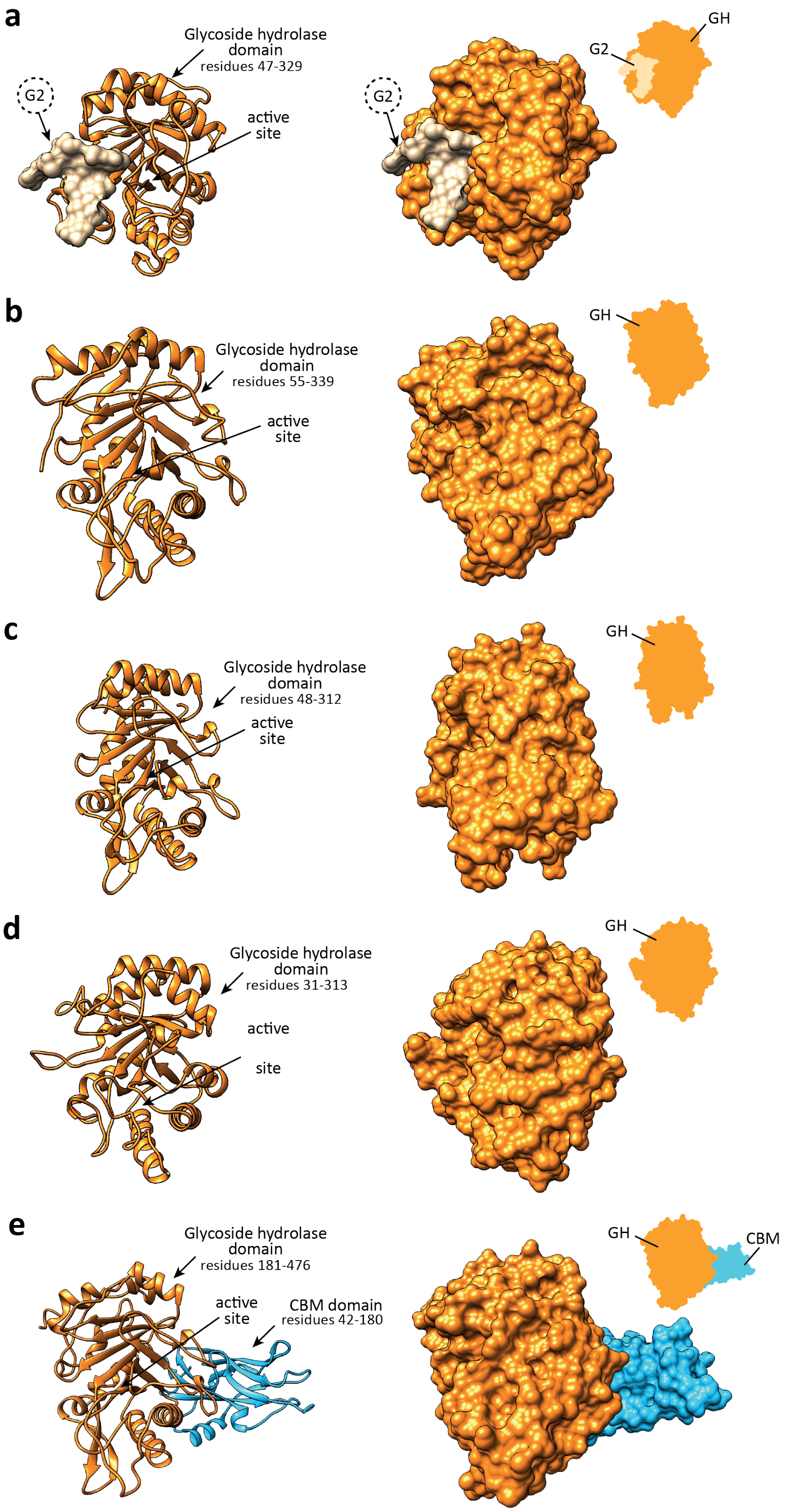

Supplementary Figure 6 Overall structure of EndoF$_3$, EndoF$_1$, EndoH, EndoT and EndoBT. Left panels: cartoon representation showing the general fold and secondary structure organization of **a** EndoF$_3$, **b** EndoF$_1$, **c** EndoH, **d** EndoT and **e** EndoBT, including the glycoside hydrolase (GH; orange), and carbohydrate binding module (cyan) domains. Right panels: surface representation of **a** EndoF$_3$, **b** EndoF$_1$, **c** EndoH, **d** EndoT and **e** EndoBT. The G2 product is shown in light brown.

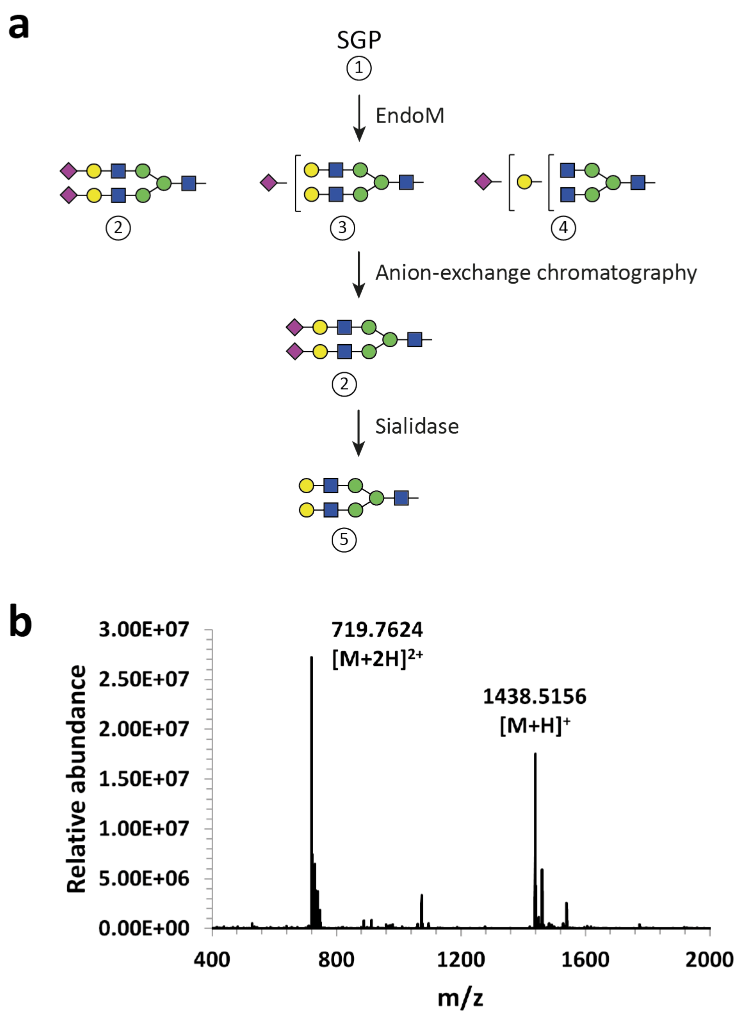

Supplementary Figure 7 Biochemical Synthesis of G2 product. **a** Scheme showing the chemical synthesis pathway of G2 product. **b** ESI Mass Spectrum of purified G2 product.

Supplementary Table 1. Data collection and refinement statistics

	$EndoS_{D233A/E235L}$-G2 product complex
**PDB code**	**6EN3**
**Beamline**	Proxima-2A
**Wavelength (Å)**	0.980105
**Resolution range (Å)**	44.07-2.9
**Space group**	$P2_1$
**Unit cell**	77.6, 90.1, 104.4, 90, 110.4, 90
**Total reflections**	118762 (17805)
**Unique reflections**	29995 (4337)
**Multiplicity**	4.0 (4.1)
**Completeness (%)**	98.8 (98.7)
**Mean I/sigma(I)**	10.6 (2.0)
**Wilson B-factor**	67.9
**R-merge**	0.096 (0.781)
**CC1/2**	0.996 (0.569)
**CC***	0.999 (0.852)
**Reflections used in refinement**	29816 (2881)
**Reflections used for R-free**	1491 (144)
**R-work**	0.2093 (0.3648)
**R-free**	0.2395 (0.3709)
**Number of non-H atoms**	7451
**Macromolecules**	7344
**Ligands**	100
**Protein residues**	934
**RMS(bonds)**	0.003
**RMS(angles)**	0.64
**Ramachandran favored (%)**	96
**Ramachandran allowed (%)**	4
**Ramachandran outliers (%)**	0
**Rotamer outliers (%)**	0.13
**Clashscore**	1.96
**Average B-factor**	71.64
**Macromolecules**	71.43
**Ligands**	87.47
**Solvent**	64.88

Statistics for the highest-resolution shell are shown in parentheses.

Supplementary Table 2. SAXS data and refinement parameters

Data collection parameters	EndoS-fl	$EndoS_{D233A/E235L}$-G2
**Instrument**	B21 (DLS)	B21 (DLS)
**Wavelength (Å)**	0.99	0.99
**S range ($Å^{-1}$)**	0.002-0.420	0.0022-0.420
**Exposure time (s per frame)**	0.56	0.56
**Concentration range (mg/ml)**	0.5-4	0.5-4
**Temperature (°C)**	25	25
**Structure parameters**		
***I*(0) (a.u.)[1] (from *P(r)*)**	0.08 ± 0.01	0.08 ± 0.01
***Rg* (Å) (from *P(r)*)**	43.2 ± 0.1	43.9 ± 0.3
***I*(0) (a.u.)[1] (from Guinier)**	0.08	0.08
***Rg* (Å) (from Guinier)**	42.6 ± 0.1	43.4 ± 0.1
**D_{max} (Å)**	139	145.5
**Porod volume estimate ($Å^3$)**	184164	186399
**Dry volume calculated from sequence ($Å^3$)**	130695	130695
**Ab initio modeling (χ^2 value)**	1.7± 0.1	1.6± 0.1
**Crysol comparison (χ^2 value)**	2.8	2.2
**SREFLEX comparison (χ^2 value)**	1.7	1.3
**Molecular mass determination**		
**Molecular mass (kDa)**	110	112
**Calculated monomeric from sequence (kDa)**	108	108.4
**Software employed**		
**Primary data reduction**	*SCÅTTER*	*SCÅTTER*
**Data processing**	*PRIMUS/SCÅTTER*	*PRIMUS/SCÅTTER*
**Ab initio analysis**	*GASBOR*	*GASBOR*
**Computation of model intensities**	*CRYSOL*	*CRYSOL*

[1] arbitrary unit
